# Low Body Mass Index Is Associated with a Long-Term Decline in Accelerometer-Measured Physical Activity in Patients with COPD: An Exploratory Analysis of Participants Co-Enrolled in Two Prospective Cohort Studies

**DOI:** 10.3390/jcm15187038

**Published:** 2026-09-11

**Authors:** Yusuke Murakami, Yoshiaki Minakata, Yasushi Tanimoto, Keisuke Miki, Shinji Tamaki, Toshiyuki Kita

**Affiliations:** 1 Department of Respiratory Medicine, NHO Wakayama Hospital, 1138 Wada, Mihama-Cho, Hidaka-Gun, Wakayama 644-0044, Japan; minakata.yoshiaki.qy@mail.hosp.go.jp; 2 Department of Respiratory Medicine, NHO Minami-Okayama Medical Center, Okayama 701-0304, Japan; tanimoto-yasushi@city.kasaoka.lg.jp; 3 Department of Respiratory Medicine, NHO Osaka Toneyama Medical Center, Osaka 560-8552, Japan; miki.keisuke.pu@mail.hosp.go.jp; 4 Department of Respiratory Medicine, NHO Nara Medical Center, Nara 630-8053, Japan; tamaki.shinji.yw@mail.hosp.go.jp; 5 Department of Respiratory Medicine, NHO Kanazawa Medical Center, Kanazawa 920-8650, Japan

**Keywords:** chronic obstructive pulmonary disease, longitudinal studies, sedentary behavior, metabolic equivalents, nutritional status

## Abstract

**Background/Objectives:** Reduced physical activity (PA) and increased sedentary behavior (SB) are associated with adverse outcomes in chronic obstructive pulmonary disease (COPD), but the factors underlying their long-term change are unclear; we investigated the baseline factors associated with these changes. **Methods:** Japanese outpatients with stable COPD co-enrolled in the SPACE (baseline) and E-PAC (follow-up) studies were analyzed retrospectively. Twenty-nine baseline clinical variables were assessed. PA and SB were measured with a waist-worn triaxial accelerometer and expressed in metabolic equivalents (METs). Annualized percentage changes (per year, relative to baseline) were analyzed by Spearman’s rank correlation and multiple linear regression. **Results:** Thirty-two participants (all men; median age 71.0 years; median follow-up 5.0 years) were analyzed. Lower baseline body weight, BMI, upper arm circumference, FEV1.0, FEV1.0/FVC, and lowest percutaneous oxygen saturation (SpO_2_) during the 6 min walk test (6MWT) were correlated with greater annualized declines in PA. For SB, the annualized change was correlated only with the lowest SpO_2_ during the 6MWT, FEV1.0/FVC, and the hemoglobin concentration. In multivariable models adjusted for FEV1.0 %pred and the lowest SpO_2_ during the 6MWT, BMI remained associated with the declines in total PA and in the duration at ≥3.0 METs; the models for the step count and SB were not significant. **Conclusions:** In this exploratory analysis, nutrition-related anthropometric measures, poorer pulmonary function, and exercise-induced desaturation were associated with the long-term decline in PA, and a lower BMI remained associated after adjustment. For the change in SB, associations were found only in unadjusted correlation analysis and were not supported by the multivariable model. These hypothesis-generating findings require confirmation in a larger cohort.

## 1. Introduction

Chronic obstructive pulmonary disease (COPD) is characterized by progressive airflow obstruction and is a major cause of morbidity and mortality worldwide [1,2]. In patients with COPD, reduced physical activity (PA) and increased sedentary behavior (SB) are strongly associated with poor outcomes and therefore have attracted increasing attention [3,4,5]. PA refers to any bodily movement produced by skeletal muscles that results in energy expenditure and it is generally evaluated based on the duration of activities performed at moderate-to-vigorous intensity (≥3 metabolic equivalents [METs]) [6,7]. In contrast, waking behaviors performed in a sitting or reclining posture with an energy expenditure of ≤1.5 METs are classified as SB [7,8].

Although numerous cross-sectional studies have investigated the factors associated with PA and SB, the factors associated with a long-term decline in PA and increase in SB remain unclear. Several longitudinal studies have demonstrated that PA progressively declines over time in patients with COPD [9,10,11], and baseline pulmonary function may influence the rate of this decline [10]. Importantly, this decline appears to occur independently of changes in exercise capacity, suggesting that behavioral and environmental factors, rather than physiological exercise capacity alone, play a substantial role [11]. However, these longitudinal studies were limited by relatively short follow-up periods, with reported mean follow-up durations of only 2.7 and 2.4 years. Furthermore, even large-scale accelerometer studies have generally assessed PA at a single time point. In a recent analysis of 1551 individuals with COPD in the UK Biobank, the authors identified the inability to capture longitudinal changes in PA as a key limitation requiring further investigation [12]. In addition, many previous studies have relied primarily on the daily step count as the sole measure of PA. Although step count is a simple and intuitive metric, it does not adequately reflect activity intensity or SB. Consequently, evidence regarding long-term changes in SB and their clinical correlates remains particularly limited.

Given the vicious cycle in which reduced PA and increased SB worsen the disease severity, thereby promoting a further decline in PA and an increase in SB, it is clinically important to identify the factors associated with a long-term decline in PA and an increase in SB [13,14]. However, longitudinal studies examining the factors associated with a long-term decline in PA and an increase in SB remain limited.

We previously conducted a cross-sectional analysis that identified exercise capacity, dyspnea, pulmonary function, exercise-induced desaturation (EID), nutritional status, muscle strength, and hemoglobin A1c (HbA1c) as factors that discriminated four activity phenotypes defined by the combination of PA and SB levels at a single time point [15]. However, whether these baseline factors are also associated with longitudinal changes in PA and SB has not been investigated. Building on these cross-sectional findings, the present study aimed to identify the baseline factors associated with a long-term decline in PA and an increase in SB over an extended follow-up period in patients with COPD. PA and SB were reassessed after an interval considerably longer than the follow-up durations reported previously, using three measures of PA together with a measure of SB rather than the step count alone, with the aim of identifying patients at risk during ordinary outpatient care.

## 2. Materials and Methods

### 2.1. Participants

Participants were recruited from the SPACE study, which developed a reference equation for PA in Japanese patients with COPD (UMIN000025459, 10 January 2017), and the E-PAC study, which developed a practical prediction equation for PA in patients with COPD (UMIN000049919, 4 January 2023). Patient enrollment in the SPACE study was conducted from January 2017 to February 2020 at 21 institutions affiliated with the National Hospital Organization (NHO) group in Japan [16]. That in the E-PAC study was conducted from October 2022 to March 2025 at 32 institutions in Japan. The baseline data for the present cohort were obtained from the SPACE study [15]. In contrast to the previous report, the present analysis is a longitudinal study using follow-up data from the E-PAC study to assess the long-term changes in PA and SB.

In the present study, the participants were outpatients with stable COPD, diagnosed based on a post-bronchodilator forced expiratory volume in one second (FEV1.0)/forced vital capacity (FVC) ratio of <0.7 and an age of ≥40 years. The exclusion criteria were as follows: (1) a history of an exacerbation within the past three months, (2) a severely reduced PA owing to other diseases (including neuromuscular disease, bone and joint disease, active malignant disease, and myocardial infarction), (3) ongoing oxygen therapy, and (4) cases in which participation in the study was deemed inappropriate by the attending physician. This investigation was designed as a retrospective longitudinal analysis that included only those who participated in both studies. The remaining SPACE participants served as the comparison group for assessing potential selection bias (Table S1), and comprised the 194 participants who were included in the analysis of the SPACE study but were not co-enrolled in the E-PAC study. Participants who were enrolled in the SPACE study but were not included in its analysis (those who did not meet the eligibility criteria of the SPACE study, those who withdrew, and those without at least three valid days of accelerometer recording) were also excluded from this comparison group; the comparison group therefore does not comprise all of the SPACE participants who were not co-enrolled in the E-PAC study.

This study was conducted in accordance with the provisions of the Declaration of Helsinki and was approved by the Ethics Committee of the NHO Wakayama Hospital (approval number: 06-1; approval date: 9 May 2024). The study was registered with the UMIN Clinical Trials Registry (UMIN000060146, 20 December 2025). The participants were notified through the websites of the hospitals where they had participated in the study and were given the opportunity to decline participation.

### 2.2. Protocol

Data from the SPACE study were used as the baseline, and data from the E-PAC study were used as the follow-up. The annualized percentage changes in PA and SB were calculated as follows: annualized percentage change = 100 × (follow-up value − baseline value)/baseline value/year. The follow-up duration in years was calculated by dividing the number of days between study registrations by 365. The percentage change rather than the absolute change was adopted as the outcome for the subsequent analyses because baseline PA and SB varied widely between participants, so that a given absolute change carries a different meaning at different baseline levels, and because the four outcomes are expressed on different scales (steps, METs·h and minutes); expressing each change relative to the participant’s own baseline places them on a common scale. Absolute annualized changes are also presented, and the correlation analysis was repeated using them as a sensitivity analysis. This quantity is referred to hereafter as the annualized change, and is expressed as a percentage of the baseline value per year. Because PA and SB were measured at only two time points, it represents the total change between baseline and follow-up divided by the follow-up interval, not a rate estimated from repeated measurements. The relationship between these changes and the baseline variables was evaluated to investigate the predisposing factors influencing long-term changes in PA and SB in patients with COPD. Information on acute exacerbations of COPD, pneumonia, and hospitalizations due to respiratory diseases occurring between the SPACE and E-PAC studies was collected by reviewing the medical records. An acute exacerbation of COPD was defined as an event requiring treatment with systemic corticosteroids and/or antibiotics, or hospitalization.

### 2.3. Variables

In the SPACE study, participants wore an accelerometer from days 0 to 14 (or up to day 28). Day 0 was defined as the day on which informed consent was obtained. On day 0, the following variables were collected: age, sex, height, body weight, body mass index (BMI), smoking history, modified Medical Research Council (mMRC) dyspnea scale, Hospital Anxiety and Depression Scale (HADS), medical history, nutritional status, grip strength, post-bronchodilation spirometry, and blood tests. The medical history included a history of COPD medication, whether or not pulmonary rehabilitation was performed, and the presence of comorbidities. The upper arm circumference and triceps subcutaneous fat thickness were measured on the dominant side. BMI was calculated as body weight (kg) divided by the square of height (m^2^). Body weight, BMI, upper arm circumference, and triceps subcutaneous fat thickness were used as anthropometric indices related to nutritional status; none of these distinguishes fat mass from fat-free or skeletal muscle mass, and no direct assessment of body composition was available in either study period. Blood tests included assessments of the red blood cell (RBC) count, hemoglobin (Hb), fasting blood glucose (FBG), HbA1c, albumin (Alb), and brain natriuretic peptide (BNP). A 6 min walk test (6MWT) was performed on day 14 (or up to day 28), during which the 6 min walk distance (6MWD) and the lowest percutaneous oxygen saturation (SpO_2_) were recorded. In the E-PAC study, participants wore the same accelerometer from days 0 to 14 (or up to day 28). Day 0 was defined as the day on which informed consent was obtained. On day 0, the following variables were collected: age, sex, height, body weight, BMI, smoking history, mMRC score, and post-bronchodilation spirometry.

### 2.4. Accelerometric Evaluation

In both studies, from day 0 to day 14 (up to day 28), PA was measured by wearing a triaxial accelerometer, Active Style Pro HJA-750C^®^ (Omron Healthcare, Kyoto, Japan), on the waist. The same device model was used in both studies, and the activity-intensity classification algorithm implemented in the device was identical at the two time points, so that the estimated METs values are directly comparable between baseline and follow-up. In the SPACE study, measurement was performed for 24 h, excluding bathing and water activities, whereas in the E-PAC study, it was performed for waking hours, also excluding bathing and water activities. Participants were required to maintain an activity diary during the accelerometer measurement period. Step count, total PA, and duration at ≥3.0 METs were employed as indicators of PA, and duration at 1.0–1.5 METs was employed as an indicator of SB. SB was therefore defined operationally as the time spent at an accelerometer-estimated intensity of 1.0–1.5 METs. This corresponds to the energy-expenditure component of the definition of SB but not to its postural component, because a waist-worn accelerometer that estimates METs cannot reliably distinguish sitting or reclining from quiet standing. Throughout this article, this variable should therefore be read as accelerometer-derived low-intensity time rather than as posture-confirmed SB.

### 2.5. Data Cleaning

First, of the 15 recorded days (up to 29 days), rainy days, days with special activities, and days when the maximum temperature was <5 °C or minimum temperature ≥ 25 °C were excluded because they were considered to affect the participants’ physical status [17,18]. Next, the accelerometric raw data from 07:00 to 20:00 were extracted from the recorded data, and the days with <8 h of accelerometer wearing time were excluded. Non-wear time was defined as 90 min of consecutive zeros with an allowance for 2 min of interruptions, in accordance with Choi’s method [19]. Finally, to obtain data reproducibility, data from participants who had at least three valid days were analyzed as valid data, and the mean of all valid days in each study period was used as the representative value for each PA and SB variable. Information on weather conditions and special activity days was obtained from the content of the diary. The maximum and minimum temperatures were retrieved from the records of the nearest weather station to each institution where the participant was recruited.

### 2.6. Sample Size and Power

The sample size was fixed by the number of participants common to both studies (*n* = 32); no a priori sample size calculation was performed, and no minimum important effect size was pre-specified for any of the associations examined. The study was therefore not powered to detect any particular magnitude of association, and the absence of statistical significance cannot be interpreted as evidence of the absence of an association. To allow readers to judge the range of effects compatible with our data, regression coefficients are reported with the 95% confidence interval (CI) for every independent variable in each model, irrespective of statistical significance. We deliberately did not perform post hoc power calculations, as these are a deterministic function of the observed *p* value and provide no information beyond these CIs.

### 2.7. Statistical Analysis

Continuous variables are presented as the median (interquartile range [IQR], Q1, Q3). Spearman’s rank correlation coefficient was used for all correlation analyses. A single non-parametric method was applied uniformly across variables, because the annualized changes were skewed, the sample size was small, and parametric coefficients are sensitive to outliers. Multiple linear regression was used, with the regression coefficients reported as unstandardized coefficients (β) with 95% CIs, to examine the association between candidate variables and the changes in PA and SB. To limit overfitting, the number of independent variables was restricted to three, corresponding to approximately 10 observations per variable. Multicollinearity was assessed using variance inflation factors (VIFs), with VIFs > 5 considered indicative of substantial multicollinearity.

All tests were two-tailed, and a threshold of *p* < 0.05 was used descriptively. The baseline characteristics of the participants included in the present analysis were compared with those of the SPACE participants who were not included (*n* = 194, defined as described above), using the Mann–Whitney U test (Table S1). Within-participant comparisons of the number of valid days and of the valid wear time between the two study periods were made using the Wilcoxon signed-rank test. Multiple linear regression analyses were performed using IBM SPSS Statistics for Windows, version 24.0 (IBM Corp., Armonk, NY, USA); all other analyses were performed using GraphPad Prism version 8.0 (GraphPad Software, San Diego, CA, USA).

### 2.8. Variable Selection for Multivariable Models

The variables entered into the models were BMI, the percentage of predicted FEV1.0 (FEV1.0 %pred), and the lowest SpO_2_ during the 6MWT. These variables were selected on the basis of their clinical relevance and their associations observed in the univariable analyses. BMI was prioritized over upper arm circumference to avoid multicollinearity; the two are strongly correlated [20,21]. BMI was selected as the representative anthropometric indicator because a low BMI has been consistently associated with the incidence, severity, and mortality of COPD in previous studies [22,23,24,25,26]. FEV1.0 %pred was employed rather than the absolute FEV1.0 value to standardize for individual differences in body size. Body weight was not entered as an alternative to BMI because it does not account for stature and therefore cannot be interpreted independently of body size, the same consideration that led us to use FEV1.0 %pred rather than the absolute FEV1.0.

### 2.9. Multiplicity

A total of 116 correlation coefficients (29 baseline variables × 4 outcome measures) and four multivariable models were computed. No adjustment for multiplicity was applied to the primary presentation of the results, nor to the between-group comparisons in Table S1. This approach was adopted because the purpose of these analyses was to identify candidate factors for subsequent confirmatory study rather than to test pre-specified hypotheses. With 116 coefficients evaluated at a two-sided threshold of 0.05, approximately 5.8 would be expected to reach this threshold by chance alone. To quantify the extent to which the observed associations might be attributable to multiple testing, the Benjamini–Hochberg procedure was applied to all 116 correlation coefficients as a post hoc sensitivity analysis, and the resulting false discovery rate-adjusted values (q values) are reported in Table S2.

Two features of these analyses mean that the figures given above understate the influence of multiplicity. First, the three variables entered into the multivariable models were selected with reference to the univariable results obtained from the same dataset; this selection is an additional source of multiplicity that is not captured by the count of 116 coefficients, and the *p* values of the multivariable models are correspondingly optimistic. Second, the coefficients are not mutually independent, since the four outcome measures were derived from the same accelerometer recordings and are strongly correlated with one another, as are several of the baseline variables. The expected number of chance findings given above is therefore an upper bound obtained under an assumption of independence, and the q values, which assume independence or positive regression dependence, should be read as a sensitivity analysis rather than as a formal guarantee of the error rate. All *p* values are presented descriptively, as indications of the strength of evidence within an exploratory analysis.

## 3. Results

**Figure 1:**
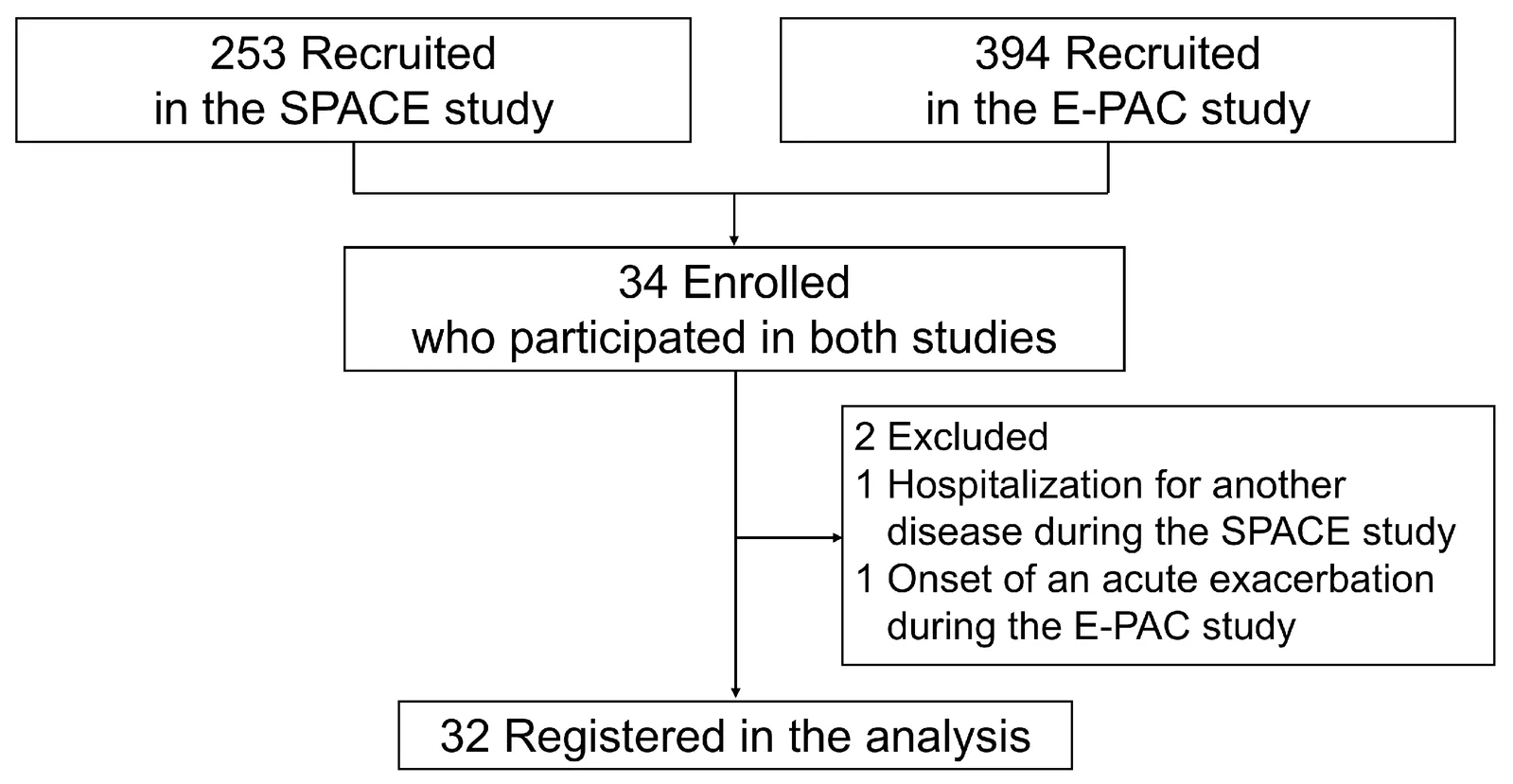
Flow diagram of participant enrollment in the present study.

Among the 253 participants enrolled in the SPACE study and the 394 enrolled in the E-PAC study, 34 took part in both. Two were excluded: one was hospitalized for another disease during the SPACE study, and the other had an acute exacerbation during the E-PAC study, thus leaving 32 participants from five institutions for analysis (Figure 1).

The median number of valid days was 12 (IQR 9–13) in the SPACE period and 10 (IQR 8–11) in the E-PAC period (*p* = 0.013), and the median valid wear time within the 07:00–20:00 window was 706 min/day (IQR 643–732) and 649 min/day (IQR 591–710), respectively (*p* = 0.002).

The median follow-up duration was 5.0 years. At baseline, the participants had a median age of 71.0 years and the median FEV1.0 %pred was 70.1%. The median daily step count, total PA, and duration at ≥3.0 METs and 1.0–1.5 METs were 4274 steps, 3.19 METs·h, 52.8 min, and 329 min, respectively. At follow-up (median age, 75.5 years), FEV1.0 %pred decreased to 65.1%. The median daily step count, total PA, and duration at ≥3.0 METs and 1.0–1.5 METs were 3679 steps, 2.43 METs·h, 39.6 min, and 349 min, respectively (Table 1).

Compared with the 194 SPACE participants who were analyzed in the SPACE study but were not co-enrolled in the E-PAC study, the 32 participants analyzed here were younger (median 71.0 vs. 74.0 years, *p* = 0.046) and had a longer 6MWD (453 vs. 400 m, *p* < 0.001). Their total PA (3.19 vs. 2.35 METs·h, *p* = 0.012) and duration at ≥3.0 METs (52.8 vs. 40.0 min, *p* = 0.014) were higher, and their duration at 1.0–1.5 METs was shorter (329 vs. 369 min, *p* = 0.010). In contrast, BMI (*p* = 0.374), FEV1.0 %pred (*p* = 0.086), and the lowest SpO_2_ during the 6MWT (*p* = 0.755) did not differ significantly between the two groups (Table S1).

**Figure 2:**
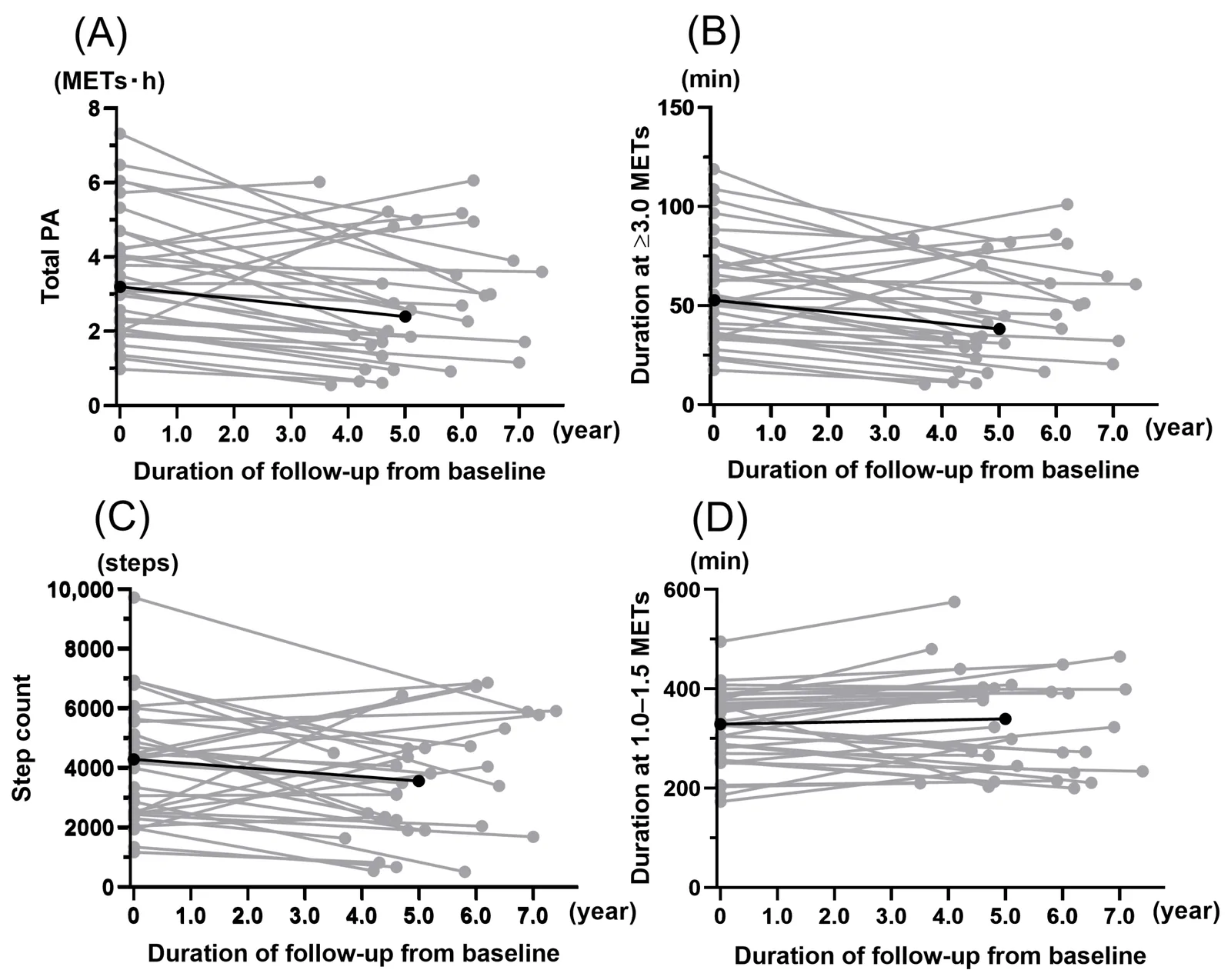
Changes in the PA and SB parameters from baseline to follow-up. (**A**) Total PA, (**B**) duration of PA at ≥3.0 METs, (**C**) step count, and (**D**) duration of SB at 1.0–1.5 METs. All values are daily values averaged over the measurement period. Gray circles represent the individual participant values, plotted at baseline (year 0) and at that participant’s own follow-up duration. Gray lines connect the baseline and the follow-up values to facilitate the visualization of changes; the intermediate values were not measured, and these lines indicate neither actual trajectories nor linear changes. The left black circle represents the median baseline value. For illustrative purposes, the right black circle was obtained by applying the median annualized change over the median follow-up period of 5.0 years to the median baseline value, and is plotted at 5.0 years; it therefore illustrates the median annualized change rather than the median of the observed follow-up values, which were obtained at differing follow-up durations. The black line represents this median annualized change applied uniformly over the median follow-up period, and is shown for illustrative purposes only. Abbreviations: PA, physical activity; SB, sedentary behavior; METs, metabolic equivalents.

**Figure 3:**
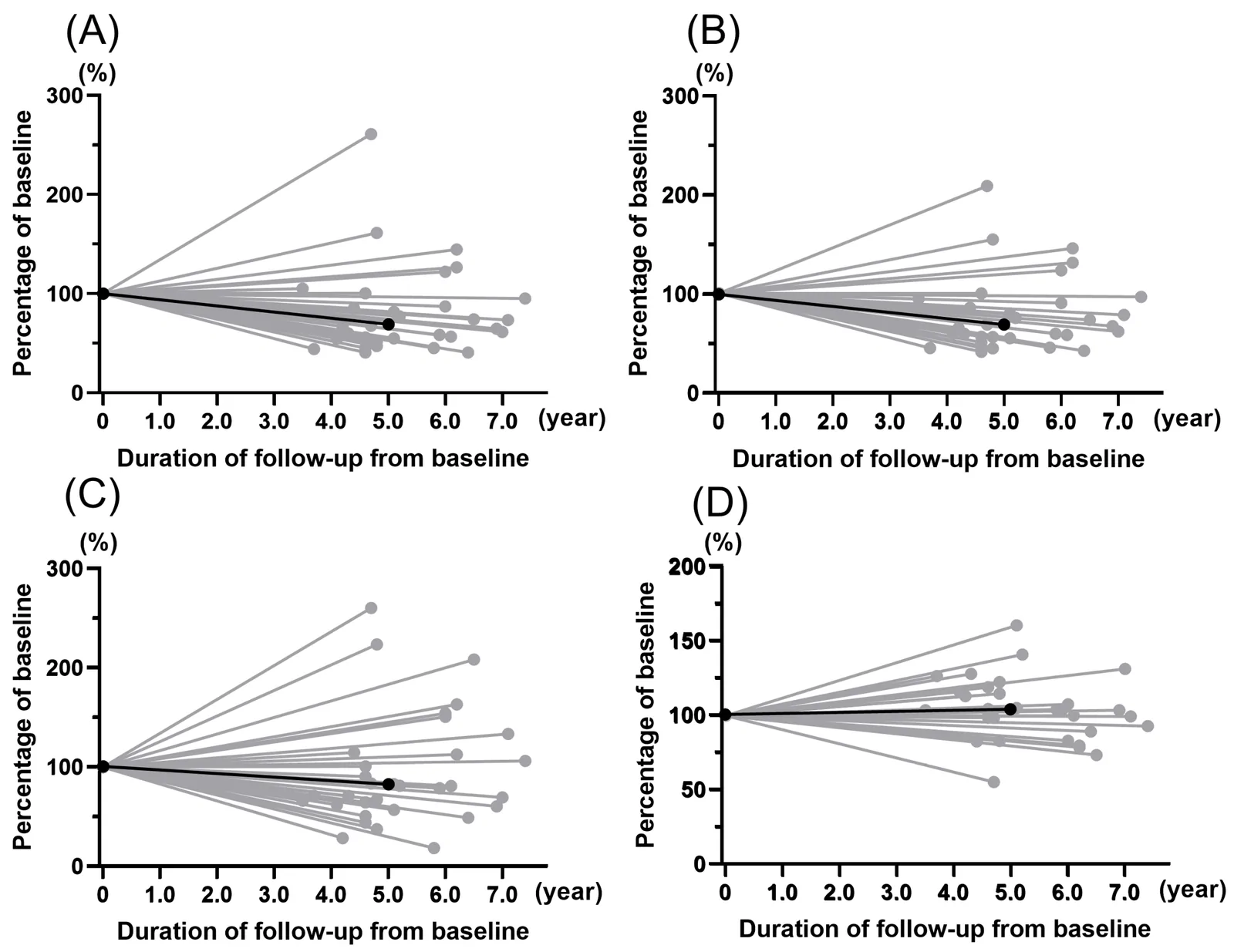
Changes in the PA and SB parameters from baseline to follow-up, expressed relative to each participant’s own baseline value. (**A**) Total PA, (**B**) duration of PA at ≥3.0 METs, (**C**) step count, and (**D**) duration of SB at 1.0–1.5 METs. Values are expressed relative to each participant’s own baseline value, which is set at 100%; a value of 100% therefore indicates no change from baseline. Gray circles represent the individual participant values, plotted at baseline (year 0) and at that participant’s own follow-up duration. Gray lines connect the baseline and the follow-up values to facilitate the visualization of changes; the intermediate values were not measured, and these lines indicate neither actual trajectories nor linear changes. The left black circle is fixed at 100% by definition. For illustrative purposes, the right black circle was obtained by applying the median annualized change, expressed as a percentage of the baseline value, over the median follow-up period of 5.0 years to the baseline value of 100%, and is plotted at 5.0 years; it therefore illustrates the median annualized change rather than the median of the observed values at follow-up. The black line represents this median annualized change applied uniformly over the median follow-up period, and is shown for illustrative purposes only. Because the median of the individual ratios is not equal to the ratio of the medians, this value does not correspond exactly to the ratio between the two black circles in Figure 2. Note that the *y*-axis scale of panel (**D**) differs from that of panels (**A**–**C**). Abbreviations: PA, physical activity; SB, sedentary behavior; METs, metabolic equivalents.

The median annualized changes in the step count, total PA, duration at ≥3.0 METs, and duration at 1.0–1.5 METs were −3.6%, −6.2%, −6.1%, and 0.7%, respectively. The median annualized changes in inspiratory capacity (IC), FVC, and FEV1.0 were −1.6%, −2.2%, and −3.0%, respectively (Table 2). Absolute changes and the annualized changes in PA and SB are illustrated in Figure 2 and Figure 3. Because PA and SB were measured at only two time points, the lines in these figures connect each participant’s baseline and follow-up values and do not represent observed longitudinal trajectories; the intermediate course is unknown. Of the 32 participants, one experienced an acute exacerbation of COPD, three developed pneumonia, and three were hospitalized due to respiratory diseases between the SPACE and E-PAC study periods.

Spearman’s rank correlation coefficients showed that the annualized changes in the step count, total PA, and duration at ≥3.0 METs were significantly positively correlated with body weight, BMI, FEV1.0, FEV1.0/FVC, lowest SpO_2_ during the 6MWT, upper arm circumference, and HADS–Anxiety subscale (HADS-A). FEV1.0 %pred was correlated with the annualized changes in the step count (r = 0.416, *p* = 0.018) and in the duration at ≥3.0 METs (r = 0.364, *p* = 0.041), but not with the change in total PA (r = 0.348, *p* = 0.051). The annualized change in the duration at 1.0–1.5 METs was negatively correlated with the lowest SpO_2_ during the 6MWT and with FEV1.0/FVC (r = −0.357, *p* = 0.045), and positively correlated with the Hb concentration, and the annualized change in the step count was negatively correlated with the RBC count (Table 3).

After application of the Benjamini–Hochberg procedure to all 116 coefficients, six remained significant at a false discovery rate of 5% (all q = 0.019): body weight with the annualized changes in the step count, total PA, and duration at ≥3.0 METs; the lowest SpO_2_ during the 6MWT with the changes in total PA and duration at ≥3.0 METs; and FEV1.0/FVC with the change in the step count. BMI (q = 0.052–0.061), upper arm circumference (q = 0.054–0.061), and FEV1.0 (q = 0.052–0.061) lay immediately above this threshold. Of the 27 coefficients that reached the conventional threshold of *p* < 0.05 before adjustment, these six were the only ones that remained significant. The remaining 21 did not, including Hb (q = 0.094) and the lowest SpO_2_ during the 6MWT (q = 0.106) with the change in the duration at 1.0–1.5 METs, FEV1.0/FVC with the change in the duration at 1.0–1.5 METs (q = 0.193), FEV1.0 %pred with the changes in the step count (q = 0.095) and in the duration at ≥3.0 METs (q = 0.183), the RBC count with the change in the step count (q = 0.130), and HADS-A with the changes in PA (q = 0.061–0.111) (Table S2).

When the correlations were repeated using the absolute annualized changes, the main findings were reproduced: the absolute declines in PA were correlated with body weight, BMI, upper arm circumference, FEV1.0/FVC and the lowest SpO_2_ during the 6MWT, and the absolute change in SB was correlated with the lowest SpO_2_ and Hb. The association with HADS-A, by contrast, was not reproduced. The absolute changes were, however, correlated with the baseline level of the same outcome, indicating that participants who were more active at baseline showed larger absolute changes.

For the annualized change in the duration at ≥3.0 METs with multiple linear regression analysis, the overall regression model was statistically significant (F = 4.31, *p* = 0.013), explaining 31.6% of the variance (adjusted R^2^ = 0.243). BMI was associated with the change in the duration at ≥3.0 METs (β = 1.07, 95% CI 0.18 to 1.97, *p* = 0.020; Table 4). For the annualized change in total PA, the model reached statistical significance (F = 3.28, *p* = 0.035), explaining 26.0% of the variance (adjusted R^2^ = 0.181). BMI was associated with the total PA change (β = 1.23, 95% CI 0.12 to 2.38, *p* = 0.031; Table 4). Regarding the annualized change in the step count, the overall model was not statistically significant (F = 2.56, *p* = 0.076), explaining 21.5% of the variance (adjusted R^2^ = 0.131). None of the independent variables were significantly associated with the step count change, although BMI showed a borderline association (β = 1.21, 95% CI −0.17 to 2.58, *p* = 0.083; Table 4). For the annualized change in the duration at 1.0–1.5 METs, the model was not statistically significant (F = 1.41, *p* = 0.259), explaining 13.2% of the variance (adjusted R^2^ = 0.039). None of the independent variables were significantly associated with the SB change (Table 4).

The VIF values were <2 for all independent variables in all models, thus indicating no evidence of problematic multicollinearity.

## 4. Discussion

We investigated the baseline factors associated with the long-term changes in PA and SB in patients with COPD. A higher body weight, BMI, upper arm circumference, FEV1.0, FEV1.0/FVC, lowest SpO_2_ during the 6MWT, and HADS-A were associated with a slower decline in the step count, total PA, and the duration at ≥3.0 METs, and a higher FEV1.0 %pred with a slower decline in the step count and in the duration at ≥3.0 METs. A higher lowest SpO_2_ during the 6MWT and a higher FEV1.0/FVC were associated with a smaller increase in SB, whereas a higher baseline Hb concentration was associated with a greater increase in SB. In the multiple linear regression analyses, BMI remained associated with the annualized changes in total PA and in the duration at ≥3.0 METs after accounting for FEV1.0 %pred and the lowest SpO_2_ during the 6MWT.

Body weight, BMI, and upper arm circumference are strongly intercorrelated and therefore do not constitute three independent observations. The stronger q value obtained for body weight does not indicate that it is the better construct: with 32 participants the difference between q = 0.019 and q = 0.052 carries little information, and body weight cannot be interpreted independently of stature. We therefore retained BMI as the representative index, where it stands for the height-standardized anthropometric signal as a whole rather than for BMI in particular. Although, to our knowledge, no studies have directly examined the relationship between nutritional status and a long-term decline in PA in patients with COPD, previous studies have shown that a low BMI is associated with an increased incidence, severity, and mortality of COPD [22,23,24,25,26]. Earlier reports have demonstrated differences in the activity levels across BMI categories in COPD populations [27,28]. Mortality due to respiratory diseases in East Asian populations is high in both the low- and high-BMI groups, which indicates that both extremely low and high BMI values may be associated with adverse outcomes [29]. A U-shaped relationship between BMI and mortality has also been reported within COPD itself, with risk rising at both the underweight and the obese extremes [30]. Mechanisms capable of impairing PA are plausible at either end. At the lower end, a reduced skeletal muscle mass, systemic inflammation, and a diminished metabolic reserve converge on limb muscle dysfunction, which is itself a principal determinant of exercise tolerance and of the activity a patient can sustain [31]. At the upper end, a high BMI is not necessarily protective for the same reasons: fat-free mass may be depleted despite a preserved or elevated BMI, and central adiposity is itself a source of low-grade systemic inflammation acting on peripheral muscle, so that muscle depletion and excess fat mass may coexist [27,32]. Obesity also raises the metabolic cost of ambulation and the ventilatory demand of exercise, which is poorly tolerated when ventilatory reserve is already constrained [28]. In the present cohort, the BMI values were generally within a relatively low-to-moderate range (median 23.1 [IQR 20.6, 24.7]), so that the participants occupy essentially the lower limb of this distribution. Therefore, the association observed between a higher BMI and a smaller annualized decline in PA may reflect an adequate nutritional status rather than a beneficial effect of excess body weight, and these data provide no information on whether the association would persist, plateau, or reverse at higher BMI values.

To determine whether these associations were driven by a small number of influential observations, we performed a sensitivity analysis using Cook’s distance. The normal probability plots and the plots of the residuals against the predicted values showed no marked departure from normality or homoscedasticity. Cook’s distance exceeded 4/n in one observation for the model of the annualized change in the duration at ≥3.0 METs and in two observations for the model of the annualized change in total PA; the maximum value was 0.39, well below the conventional threshold of 1. After exclusion of these observations, both models remained significant (≥3.0 METs: F = 6.05, *p* = 0.003, adjusted R^2^ = 0.335; total PA: F = 4.61, *p* = 0.010, adjusted R^2^ = 0.272), and BMI remained associated with both outcomes (≥3.0 METs: β = 0.80, 95% CI 0.13 to 1.47, *p* = 0.022; total PA: β = 0.99, 95% CI 0.12 to 1.85, *p* = 0.027). The coefficients were smaller than in the full models but lay within their CIs, indicating that these associations were not driven by a small number of influential observations.

Although the observed associations appeared robust in sensitivity analyses, the biological interpretation of BMI requires caution. Taken together, these previous reports and the mechanisms outlined above provide only indirect evidence that BMI may be associated with the annualized decline in PA. Because those mechanisms act through limb muscle dysfunction rather than through body mass itself [31], a low BMI is more plausibly a readily available surrogate for that constellation than a cause in its own right [27,33], and may therefore be useful as an early marker for identifying patients at risk of an accelerated decline in PA. This interpretation is nevertheless constrained by what was measured. Neither the fat-free mass nor the skeletal muscle mass was assessed, and BMI cannot separate these compartments; sarcopenia, a plausible mediator of the association observed here, could not be evaluated in either study, since muscle quality was not measured and grip strength alone is insufficient to establish the diagnosis. Any account of the present findings in terms of sarcopenia or of fat-free mass depletion is therefore a mechanistic rationale rather than evidence of causation. The present analysis is observational and establishes only that baseline BMI and the subsequent annualized change in PA are associated; it cannot determine whether a low BMI drives the decline in PA, whether a declining activity level itself erodes muscle mass and body weight, or whether both reflect a shared underlying process such as progressive systemic involvement of the disease.

Clinically, the appeal of these anthropometric indices lies in their accessibility. BMI and upper arm circumference require no specialized equipment and can be obtained at a routine outpatient visit alongside spirometry and symptom scores, and body composition is already recognized as a component of nutritional assessment in COPD. If the association observed here is confirmed, these indices could help to identify patients whose PA is likely to decline more rapidly and who may therefore warrant closer monitoring of their activity. These indices identify risk; they do not indicate treatment. The present data cannot establish that improving nutritional status or raising BMI would slow the decline in PA, and whether nutritional or exercise interventions alter its subsequent trajectory is a question for a prospective interventional study.

A higher lowest SpO_2_ during the 6MWT was associated with a smaller annualized decline in PA and a smaller increase in SB. EID is a marker of disease severity in COPD and has been correlated with an increased risk of mortality [34,35] and a greater impairment of pulmonary function [34]. A cross-sectional study reported that EID was associated with impaired daily PA in COPD [36]. Although no longitudinal studies have directly examined the associations between EID and a long-term decline in PA or an increase in SB, EID has been identified as a predictor of changes in exercise capacity [37]. These findings support the hypothesis that the physiological mechanisms underlying EID may also contribute to a long-term decline in PA and an increase in SB. The association with SB, however, rests on the univariable analysis alone: the multivariable model for the change in SB was not statistically significant, and this association did not remain significant after adjustment for the false discovery rate (q = 0.106), whereas the corresponding associations with the changes in total PA and in the duration at ≥3.0 METs did (Table S2). The finding for SB is therefore the weaker of the two.

Hypoxemia may attenuate the beneficial effects of exercise on skeletal muscles in patients with COPD [38]. Repeated desaturation can impair oxygen delivery to the peripheral muscles and promote skeletal muscle dysfunction, which may contribute to the progressive decline in PA [31,39]. In addition, pulmonary hypertension related to chronic hypoxemia may also contribute to a reduced exercise capacity in COPD [40,41]. However, this could not be evaluated in the present study because no echocardiographic or hemodynamic data were available.

In the present study, a higher baseline FEV1.0 was associated with a smaller annualized decline in PA over the follow-up period. This finding is consistent with a previous prospective study that identified pulmonary function as an important determinant of lower long-term PA levels [42]. Cross-sectional studies have reported that PA in COPD is related to pulmonary and systemic disease components, although airflow limitation alone explains only a limited part of the variance, with dynamic hyperinflation and extrapulmonary factors playing a larger role [43,44]. However, longitudinal evidence on whether baseline pulmonary function is associated with the subsequent annualized decline in PA, rather than with PA levels alone, remains scarce. The present study addresses this gap by showing that, over a 5-year follow-up period, a higher baseline pulmonary function was associated with a smaller annualized decline in PA, thereby extending previous, largely cross-sectional observations to a longitudinal setting. After control of the false discovery rate, however, only the correlation between FEV1.0/FVC and the change in the step count remained significant, whereas those for FEV1.0 (q = 0.052–0.061) and FEV1.0 %pred (q = 0.095–0.211) did not; the association between baseline pulmonary function and the subsequent change in PA should therefore be regarded as suggestive rather than established.

A positive correlation was observed between HADS-A and the annualized change in PA. However, previous studies have reported inconsistent associations between anxiety and PA in COPD [45,46,47]. Moreover, in the present cohort the anxiety levels were generally low, and the association did not remain significant after adjustment for the false discovery rate (q = 0.061–0.111; Table S2). Anxiety was moreover assessed with a single instrument; the lack of corroboration by a second measure reflects the measurement design rather than evidence against the association. This finding is exploratory and may well have arisen by chance, confirming it would require a larger sample and a hypothesis specified in advance.

A positive correlation was also observed between the baseline Hb and the annualized change in the duration at 1.0–1.5 METs, indicating that a higher Hb was associated with a greater increase in SB. A parallel finding was obtained for the RBC count, which was negatively correlated with the annualized change in the step count (r = −0.389, *p* = 0.028), indicating that a higher RBC count was associated with a greater decline in the step count. Because the RBC count and Hb are closely collinear, these two coefficients are unlikely to represent independent associations. The same directional pattern was in fact present for both markers across both outcomes, with each reaching the conventional threshold for only one of them (RBC count and the change in the duration at 1.0–1.5 METs, r = 0.344, *p* = 0.054; Hb and the change in the step count, r = −0.307, *p* = 0.087). We therefore interpret them as a single signal related to the red cell mass rather than as two separate observations. The Hb concentrations and RBC counts in this cohort were within the normal range in most participants, so that these associations reflect neither anemia nor polycythemia; furthermore, neither Hb nor the RBC count was correlated with the lowest SpO_2_ during the 6MWT, which would have suggested a shared relationship with hypoxemia. Neither association remained significant after adjustment for the false discovery rate (Hb and the change in the duration at 1.0–1.5 METs, q = 0.094; RBC count and the change in the step count, q = 0.130; Table S2). Given the absence of any accompanying abnormality in the red cell indices, and the lack of a relationship with hypoxemia, we regard the red cell findings as chance observations, and we draw no clinical inference from them here. Whether a higher red cell mass within the normal range nevertheless carries information about the subsequent trajectory of PA and SB in COPD remains an open question for future investigation in a larger cohort with prespecified hypotheses.

Our previous cross-sectional analysis of the baseline SPACE cohort demonstrated that nutritional indicators (BMI and upper arm circumference), muscle strength, pulmonary function, and EID were factors that discriminated the activity phenotypes at a single time point [15]. The present longitudinal study extends those findings by demonstrating that several of these baseline factors, particularly BMI and the lowest SpO_2_ during the 6MWT, not only characterize the current activity pattern but are also associated with the annualized decline in PA and the annualized increase in SB. Notably, the present analysis suggests that BMI may be associated with the subsequent annualized decline in PA, a relationship that cannot be examined in a cross-sectional design, and this represents the principal contribution of the present study. It remains a hypothesis to be tested in an adequately powered prospective cohort.

Our study has several limitations. First, the analysis was exploratory throughout: no power calculation was performed, no baseline factor was designated as being of primary interest in advance, the prominence given to BMI was decided after the results had been obtained, and the three variables entered into the regression models were selected with reference to the univariable results from the same dataset, so the *p* values from these models may be optimistically biased. Some of the CIs we report are wide and do not exclude clinically meaningful differences, so non-significant findings should not be interpreted as evidence of absence, and of the 116 correlation coefficients evaluated, only six remained significant after adjustment for the false discovery rate (Table S2). The associations described here are therefore candidate hypotheses, and independent replication in an adequately powered cohort is required before they can be considered established. Second, PA and SB were measured on only two occasions, and the annualized change was obtained by dividing the total change between them by the follow-up interval, which presupposes an approximately constant rate of change. The intervening trajectory is unknown and non-linear change cannot be detected in these data, so this metric summarizes the difference between two measurements on a per-year scale rather than an observed rate of decline. Because it was expressed relative to the baseline value, measurement error in the baseline contributes both to the denominator and to the outcome; the absolute annualized changes were correlated with the corresponding baseline values in the same way, so neither metric is free of this dependence and some regression to the mean cannot be excluded. Third, PA in COPD is shaped over a 5-year interval by exacerbations, hospitalizations, pulmonary rehabilitation, changes in pharmacological treatment, incident comorbidities, and changes in body weight and pulmonary function, of which only COPD exacerbations and hospitalization for other respiratory disease were recorded, and the baseline and follow-up assessments were not deliberately matched for season. The time-dependent modeling that would be required to separate these influences is uninterpretable with 32 participants, so our models cannot distinguish whether baseline BMI is itself related to the later decline or whether it marks the disease characteristics and the clinical course that followed; substantial residual confounding should be assumed. Fourth, the accelerometer wear protocol differed between the two time points: 24 h wear in the SPACE study and waking-hours wear in the E-PAC study. Extracting the 07:00–20:00 window, consistent with the recommendation of the European Respiratory Society task force that PA be assessed over waking hours [14], and processing both datasets identically thereafter were intended to make the recordings comparable, but the difference cannot be assumed to have been removed, because Choi’s algorithm was developed for waking-wear data and more readily classifies daytime sleep and prolonged quiet rest within a 24 h recording as non-wear [19], and because the instructions given to participants may themselves have affected wear behavior. Both the number of valid days and the valid wear time were lower in the E-PAC period, although both medians exceed the three valid days reported to be sufficient for reproducible estimates with this device [17]. SB is the outcome most exposed to this problem, being defined by counts at 1.0–1.5 METs, the intensity range closest to the threshold that drives that classification; posture was also not recorded, so the variable analyzed here represents accelerometer-derived low-intensity time rather than posture-confirmed sedentary behavior as currently defined. As SB was the only outcome to show a positive median annualized change (+0.7%), with an interquartile range that includes zero, we cannot exclude the possibility that this increase is partly a measurement artifact, and the SB findings require confirmation in a cohort using an identical wear protocol at both time points. Fifth, patients who died, deteriorated clinically, or were unable to complete repeated monitoring were necessarily excluded, and participation in both studies is likely to have favored cooperative and comparatively active individuals; consistent with this, comparison with the SPACE participants who were not included (Table S1) showed a better preserved exercise capacity, a higher level of PA, and a shorter duration of SB in the present cohort, which therefore represents a relatively mild, well-preserved, and physically active subgroup of the COPD population, a restricted range that may have attenuated the associations examined here. All participants were also Japanese men. PA and SB patterns differ by sex [48,49] and are strongly influenced by environmental and lifestyle factors such as weather, season, and employment status [50], which shape not only the amount of activity but the way in which it is accumulated: in people with COPD, awake sedentary time and moderate-to-vigorous activity differ between countries even after adjustment for disease severity [51], and culturally specific patterns of daily living may operate in directions that are not intuitive, such as older Japanese adults who drive being less sedentary and more physically active than those who do not [52]. Generalizability to women and to populations whose daily activity is structured differently is therefore limited. Sixth, all follow-up assessments were performed during or after the COVID-19 pandemic and all baseline assessments before it. Japan issued repeated requests for voluntary restraint of outings and social activity rather than a legally enforced lockdown, and a reduction in PA among community-dwelling older adults during the first year of the pandemic has been reported [53]. The cohort therefore contains no contemporaneous unexposed comparison group, a period effect cannot be separated from disease progression and aging, and the magnitude of the decline may be overestimated relative to what would be expected under ordinary circumstances. Taken together, the present findings should be regarded as exploratory and hypothesis-generating, and their generalizability requires confirmation in larger and more diverse cohorts.

## 5. Conclusions

In conclusion, PA showed a smaller annualized decline in participants with COPD who had higher anthropometric indices, a preserved pulmonary function, and a higher lowest SpO_2_ during the 6MWT. In the multivariable analysis, a lower BMI was still associated with a greater annualized decline in total PA and in the duration at ≥3.0 METs after adjustment for FEV1.0 %pred and the lowest SpO_2_ during the 6MWT. In contrast, no baseline variable was associated with the increase in SB after correction for multiple comparisons, and the corresponding multivariable model was not significant. A low BMI may therefore serve as an accessible clinical indicator for identifying patients whose PA warrants closer monitoring. Whether nutritional or exercise interventions modify the subsequent trajectory of PA was not addressed by this study and remains to be examined in prospective interventional research.

## Figures and Tables

**Table 1:** Characteristics of the participants included in the analysis.

	Baseline (the SPACE Study)	Follow-Up (the E-PAC Study)
**Sex** (Male/Female)	32/0	
**Follow-up duration** (years)		5.0 (4.6, 6.1)
**Age** (years)	71.0 (67.8, 75.3)	75.5 (73.8, 81.0)
**Height** (cm)	168.0 (161.6, 171.0)	168.1 (161.6, 170.9)
**Weight** (kg)	63.7 (55.6, 69.6)	63.5 (53.8, 68.8)
**BMI** (kg/m^2^)	23.1 (20.6, 24.7)	23.3 (20.4, 24.3)
**Smoking history**		
Current/Former/Never	3/29/0	2/30/0
Pack-year	47.5 (37.3, 85.5)	53.0 (39.8, 80.0)
**mMRC** (0/1/2/3/4)	7/21/2/2/0	1/22/5/3/1
**GOLD grade** (1/2/3/4)	9/17/5/1	6/18/6/2
**Pulmonary function**		
IC (L)	2.46 (2.18, 2.79)	2.27 (1.80, 2.63)
FVC (L)	3.55 (3.12, 3.82)	3.16 (2.69, 3.49)
FVC %pred (%)	101.7 (94.2, 111.6)	90.9 (80.5, 106.2)
FEV1.0 (L)	1.98 (1.53, 2.17)	1.66 (1.17, 2.05)
FEV1.0 %pred (%)	70.1 (58.5, 83.8)	65.1 (49.0, 73.6)
FEV1.0/FVC (%)	55.2 (48.6, 64.0)	54.5 (42.3, 60.8)
**6MWD** (m)	453 (417, 503)	
**Lowest SpO_2_ during the 6MWT** (**%**)	90.0 (87.0, 91.5)	
**PA**		
Step count (steps)	4274 (2492, 5262)	3679 (2036, 4903)
Total PA (METs·h)	3.19 (2.06, 4.36)	2.43 (1.57, 3.69)
Duration at ≥3.0 METs (min)	52.8 (36.1, 70.7)	39.6 (27.6, 62.1)
**SB**		
Duration at 1.0–1.5 METs (min)	329 (267, 378)	349 (242, 400)
**Nutrition**		
Upper arm circumference (cm)	28.4 (26.6, 29.8)	
Subcutaneous fat thickness of triceps brachii (cm)	0.90 (0.60, 1.20)	
**Grip strength** (kg)	35.0 (33.8, 39.3)	
**Blood tests**		
FBG (mg/dL)	102 (95, 111)	
HbA1c (%)	5.9 (5.7, 6.2)	
RBC (×10^4^/μL)	479 (442, 515)	
Hb (g/dL)	14.9 (14.3, 15.7)	
BNP (pg/mL)	17.0 (6.4, 46.9)	
Alb (g/dL)	4.3 (4.1, 4.4)	
**HADS**		
Anxiety score	3.0 (0.8, 6.0)	
Depression score	2.5 (1.0, 5.3)	
**Treatment** (Yes/No)	28/4	
**Rehabilitation** (Yes/No)	3/29	
**Comorbidities** (Yes/No)	22/10	

Continuous variables are expressed as the median (interquartile range, Q1, Q3), and categorical variables as the number of participants. Abbreviations: BMI, body mass index; mMRC, modified Medical Research Council dyspnea scale; GOLD, Global Initiative for Chronic Obstructive Lung Disease; IC, inspiratory capacity; FVC, forced vital capacity; FEV1.0, forced expiratory volume in one second; %pred, % of predicted value; 6MWD, 6 min walk distance; SpO_2_, percutaneous oxygen saturation; 6MWT, 6 min walk test; PA, physical activity; METs, metabolic equivalents; SB, sedentary behavior; FBG, fasting blood glucose; HbA1c, hemoglobin A1c; RBC, red blood cell count; Hb, hemoglobin; BNP, brain natriuretic peptide; Alb, albumin; HADS, hospital anxiety and depression scale.

**Table 2:** Changes in physical activity, sedentary behavior, and pulmonary function.

	Annualized Absolute Change	Annualized Percentage Change
**PA**		
Step count (steps)	−145 (−372, 84)	−3.6 (−8.0, 2.4)
Total PA (METs·h)	−0.16 (−0.29, −0.05)	−6.2 (−9.4, −1.8)
Duration at ≥3.0 METs (min)	−2.9 (−4.9, −1.0)	−6.1 (−9.4, −1.6)
**SB**		
Duration at 1.0–1.5 METs (min)	2.1 (−3.0, 10.4)	0.7 (−1.1, 3.1)
**Pulmonary function**		
IC (L)	−0.044 (−0.088, 0.004)	−1.6 (−3.8, 0.2)
FVC (L)	−0.067 (−0.105, −0.025)	−2.2 (−3.0, −0.7)
FEV1.0 (L)	−0.056 (−0.072, −0.026)	−3.0 (−4.5, −1.3)

The results are expressed as median (interquartile range, Q1, Q3). Abbreviations: PA, physical activity; METs, metabolic equivalents; SB, sedentary behavior; IC, inspiratory capacity; FVC, forced vital capacity; FEV1.0, forced expiratory volume in one second.

**Table 3:** Correlations between the baseline characteristics and the annualized changes in physical activity and sedentary behavior.

	Annualized Change in Step Count		Annualized Change in Total PA		Annualized Change in Duration of PA at ≥3.0 METs		Annualized Change in Duration of SB at 1.0–1.5 METs	
	r	*p*	r	*p*	r	*p*	r	*p*
**Age** (**years**)	0.234	0.197	−0.200	0.272	−0.173	0.345	−0.099	0.590
**Height** (**cm**)	0.033	0.858	−0.031	0.866	0.033	0.858	0.176	0.335
**Weight** (**kg**)	0.550	0.001	0.606	<0.001	0.590	<0.001	−0.258	0.154
**BMI** (**kg/m^2^**)	0.461	0.008	0.491	0.004	0.470	0.007	−0.278	0.124
**Smoking history** (**pack-year**)	0.047	0.800	0.030	0.872	0.007	0.971	0.296	0.100
**Pulmonary function**								
IC (L)	0.152	0.406	0.297	0.099	0.291	0.107	−0.142	0.438
FVC (L)	0.065	0.723	0.228	0.209	0.205	0.260	0.220	0.227
FVC %pred (%)	0.055	0.767	0.115	0.532	0.097	0.598	0.121	0.509
FEV1.0 (L)	0.449	0.010	0.486	0.005	0.496	0.004	−0.141	0.442
FEV1.0/FVC (%)	0.556	0.001	0.451	0.010	0.483	0.005	−0.357	0.045
FEV1.0 %pred (%)	0.416	0.018	0.348	0.051	0.364	0.041	−0.162	0.376
**6MWD** (m)	0.338	0.059	0.294	0.103	0.322	0.072	−0.115	0.531
**Lowest SpO_2_ during the 6MWT** (**%**)	0.493	0.004	0.577	<0.001	0.558	<0.001	−0.406	0.021
**Nutrition**								
Upper arm circumference (cm)	0.447	0.010	0.475	0.006	0.479	0.006	−0.286	0.112
Subcutaneous fat thickness of triceps brachii (cm)	0.060	0.744	0.135	0.462	0.146	0.427	−0.223	0.220
**Grip strength** (**kg**)	0.329	0.066	0.307	0.087	0.279	0.123	−0.127	0.488
**Blood tests**								
FBG (mg/dL)	0.058	0.753	−0.022	0.905	−0.023	0.900	0.150	0.413
HbA1c (%)	−0.025	0.894	0.190	0.297	0.162	0.376	0.020	0.912
RBC (×10^4^/μL)	−0.389	0.028	−0.154	0.399	−0.166	0.364	0.344	0.054
Hb (g/dL)	−0.307	0.087	−0.272	0.133	−0.282	0.118	0.420	0.017
BNP (pg/mL)	0.250	0.167	0.096	0.602	0.069	0.709	−0.302	0.093
Alb (g/dL)	−0.043	0.817	0.225	0.217	0.248	0.172	0.001	0.994
**HADS**								
Anxiety score	0.402	0.023	0.449	0.010	0.445	0.011	−0.305	0.089
Depression score	0.197	0.280	0.208	0.254	0.230	0.206	−0.229	0.208
**mMRC**	−0.145	0.429	0.003	0.985	−0.021	0.910	−0.041	0.826
**PA**								
Step count (steps)	−0.135	0.462	−0.031	0.866	−0.000	0.998	0.077	0.675
Total PA (METs·h)	0.205	0.261	0.169	0.356	0.194	0.287	−0.088	0.632
Duration at ≥3.0 METs (min)	0.205	0.261	0.157	0.390	0.180	0.324	−0.092	0.616
**SB**					min			
Duration at 1.0–1.5 METs (min)	−0.157	0.390	−0.215	0.238	−0.193	0.290	0.048	0.796

All coefficients represent Spearman’s rank correlation between the baseline variable and the annualized change in the corresponding outcome, expressed as a percentage of the baseline value per year. False discovery rate-adjusted q values for all 116 coefficients are given in Table S2. Because PA declined and SB increased over follow-up, the direction indicating a favorable trajectory differs between columns: in the three PA columns a positive coefficient denotes a slower decline in PA, whereas in the SB column a positive coefficient denotes a greater increase in SB. Abbreviations: BMI, body mass index; mMRC, modified Medical Research Council dyspnea scale; IC, inspiratory capacity; FVC, forced vital capacity; FEV1.0, forced expiratory volume in one second; %pred, % of predicted value; 6MWD, 6 min walk distance; 6MWT, 6 min walk test; SpO_2_, percutaneous oxygen saturation; FBG, fasting blood glucose; HbA1c, hemoglobin A1c; RBC, red blood cell count; Hb, hemoglobin; BNP, brain natriuretic peptide; Alb, albumin; HADS, hospital anxiety and depression scale; PA, physical activity; METs, metabolic equivalents; SB, sedentary behavior.

**Table 4:** Multiple linear regression analysis of the baseline factors associated with the annualized changes in physical activity and sedentary behavior.

	Annualized Change in Step Count		Annualized Change in Total PA		Annualized Change in Duration at ≥3.0 METs		Annualized Change in Duration at 1.0–1.5 METs	
	β (95% CI)	*p*	β (95% CI)	*p*	β (95% CI)	*p*	β (95% CI)	*p*
**BMI** (kg/m^2^)	1.21 (−0.17, 2.58)	0.083	1.23 (0.12, 2.38)	0.031	1.07 (0.18, 1.97)	0.020	−0.41 (−0.95, 0.13)	0.127
**FEV1.0 %pred** (%)	−0.003 (−0.25, 0.24)	0.979	−0.07 (−0.27, 0.13)	0.480	−0.05 (−0.21, 0.11)	0.519	0.03 (−0.07, 0.13)	0.508
**Lowest SpO_2_ during the 6MWT** (%)	0.41 (−0.38, 1.21)	0.295	0.45 (−0.19, 1.09)	0.164	0.44 (−0.08, 0.95)	0.094	−0.14 (−0.45, 0.17)	0.359

β, unstandardized regression coefficient; CI, confidence interval. Abbreviations: PA, physical activity; METs, metabolic equivalents; BMI, body mass index; FEV1.0, forced expiratory volume in one second; %pred, % of predicted value; 6MWT, 6 min walk test; SpO_2_, percutaneous oxygen saturation.

## Data Availability

The data presented in this study are available on request from the corresponding author owing to ethical and privacy restrictions.

## Supplementary Materials

The following supporting information can be downloaded at: https://www.mdpi.com/article/10.3390/jcm15187038/s1, Table S1: Comparison of the baseline characteristics between the participants included in the present analysis and the other participants in the SPACE study. Table S2: False discovery rate-adjusted q values for the correlations between baseline characteristics and the annualized changes in physical activity and sedentary behavior.
