# Supplementary material for: Low Body Mass Index Is Associated with a Long-Term Decline in Accelerometer-Measured Physical Activity in Patients with COPD: An Exploratory Analysis of Participants Co-Enrolled in Two Prospective Cohort Studies
Source: J Clin Med. 2026 Sep 11;15(18):7038. doi: 10.3390/jcm15187038 (PMC13607005; doi:10.3390/jcm15187038)
Supplement: Supplementary file 1 [file jcm-15-07038-s001.zip › jcm-4539525-supplementary.pdf]

Table S1. Comparison of the baseline characteristics between the participants included in the present analysis and the other participants in the SPACE study.

	Included in the present analysis (n = 32)	Analyzed in the SPACE study but not co-enrolled in E-PAC (n = 194)	p
**Age** (years)	71.0 (67.8, 75.3)	74.0 (69.0, 78.0)	0.046
**BMI** (kg/m^2)	23.1 (20.6, 24.7)	22.7 (20.2, 24.8)	0.374
**GOLD grade** (1/2/3/4)	9/17/5/1	44/93/43/14	
**FEV1.0 %pred** (%)	70.1 (58.5, 83.8)	60.9 (46.5, 78.6)	0.086
**6MWD** (m)	453 (417, 503)	400 (340, 456)	<0.001
**Lowest SpO_2 during the 6MWT (%)**	90.0 (87.0, 91.5)	90.0 (86.0, 93.0)	0.755
**PA**			
Step count (steps)	4274 (2492, 5262)	3244 (1753, 4847)	0.076
Total PA (METs·h)	3.19 (2.06, 4.36)	2.35 (1.44, 3.81)	0.012
Duration at ≥3.0 METs (min)	52.8 (36.1, 70.7)	40.0 (24.3, 63.0)	0.014
**SB**			
Duration at 1.0–1.5 METs (min)	329 (267, 378)	369 (297, 445)	0.010

The results are expressed as median (interquartile range, Q1, Q3), except for the GOLD grade, which is expressed as the number of patients.

The comparison group comprises the 194 participants who were analyzed in the SPACE study but were not co-enrolled in the E-PAC study; it therefore does not comprise all of the SPACE participants who were not co-enrolled (see Methods).

Continuous variables were compared using the Mann–Whitney U test; the p-values are unadjusted and descriptive. The distribution of the GOLD grade is shown descriptively and was not formally tested.

Abbreviations: BMI, body mass index; GOLD, Global Initiative for Chronic Obstructive Lung Disease; FEV1.0, forced expiratory volume in one second; %pred, % of predicted value; 6MWD, 6-minute walk distance; 6MWT, 6-minute walk test; SpO_2, percutaneous oxygen saturation; PA, physical activity; METs, metabolic equivalents; SB, sedentary behavior.

Table S2. False discovery rate–adjusted q-values for the correlations between baseline characteristics and the annualized changes in physical activity and sedentary behavior.

	Annualized change in step count		Annualized change in total PA		Annualized change in duration at ≥3.0 METs		Annualized change in duration at 1.0–1.5 METs	
	**r**	**q**	**r**	**q**	**r**	**q**	**r**	**q**
**Age (years)**	0.234	0.466	-0.200	0.517	-0.173	0.589	-0.099	0.776
**Height (cm)**	0.033	0.945	-0.031	0.945	0.033	0.945	0.176	0.580
**Weight (kg)**	0.550	0.019	0.606	0.019	0.590	0.019	-0.258	0.388
**BMI (kg/m²)**	0.461	0.061	0.491	0.052	0.470	0.058	-0.278	0.327
**Smoking history (pack-year)**	0.047	0.928	0.030	0.945	0.007	0.997	0.296	0.305
**Pulmonary function**								
IC (L)	0.152	0.620	0.297	0.305	0.291	0.310	-0.142	0.633
FVC (L)	0.065	0.883	0.228	0.466	0.205	0.505	0.220	0.479
FVC %pred (%)	0.055	0.908	0.115	0.709	0.097	0.776	0.121	0.695
FEV1.0 (L)	0.449	0.061	0.486	0.053	0.496	0.052	-0.141	0.633
FEV1.0/FVC (%)	0.556	0.019	0.451	0.061	0.483	0.053	-0.357	0.193
FEV1.0 %pred (%)	0.416	0.095	0.348	0.211	0.364	0.183	-0.162	0.606
**6MWD (m)**	0.338	0.228	0.294	0.306	0.322	0.261	-0.115	0.709
**Lowest SpO_2 during the 6MWT (%)**	0.493	0.052	0.577	0.019	0.558	0.019	-0.406	0.106
**Nutrition**								
Upper arm circumference (cm)	0.447	0.061	0.475	0.054	0.479	0.054	-0.286	0.317
Subcutaneous fat thickness of triceps brachii (cm)	0.060	0.899	0.135	0.646	0.146	0.630	-0.223	0.473
**Grip strength (kg)**	0.329	0.247	0.307	0.295	0.279	0.327	-0.127	0.674
**Blood tests**								
FBG (mg/dL)	0.058	0.900	-0.022	0.945	-0.023	0.945	0.150	0.622
HbA1c (%)	-0.025	0.945	0.190	0.530	0.162	0.606	0.020	0.945
RBC ($\times 10^4$/μl)	-0.389	0.130	-0.154	0.617	-0.166	0.603	0.344	0.216

	Annualized change in step count		Annualized change in total PA		Annualized change in duration at ≥3.0 METs		Annualized change in duration at 1.0–1.5 METs	
	**r**	**q**	**r**	**q**	**r**	**q**	**r**	**q**
Hb (g/dL)	-0.307	0.295	-0.272	0.343	-0.282	0.326	0.420	0.094
BNP (pg/mL)	0.250	0.412	0.096	0.776	0.069	0.875	-0.302	0.300
Alb (g/dL)	-0.043	0.938	0.225	0.473	0.248	0.416	0.001	0.998
**HADS**								
Anxiety score	0.402	0.111	0.449	0.061	0.445	0.064	-0.305	0.295
Depression score	0.197	0.524	0.208	0.505	0.230	0.466	-0.229	0.466
**mMRC**	-0.145	0.630	0.003	0.998	-0.021	0.945	-0.041	0.939
**PA**								
Step count (steps)	-0.135	0.646	-0.031	0.945	0.000	0.998	0.077	0.842
Total PA (METs·h)	0.205	0.505	0.169	0.598	0.194	0.526	-0.088	0.797
Duration at ≥3.0 METs (min)	0.205	0.505	0.157	0.611	0.180	0.569	-0.092	0.785
**SB**								
Duration at 1.0–1.5 METs (min)	-0.157	0.611	-0.215	0.493	-0.193	0.526	0.048	0.928

r, Spearman's rank correlation coefficient; q, p-value adjusted for the false discovery rate. Annualized changes are expressed as a percentage of the baseline value per year. The Benjamini–Hochberg procedure was applied to all 116 correlations shown in Table 3 (29 baseline variables × 4 outcomes). Coefficients with $q < 0.05$ are shown in bold. Unadjusted p-values are given in Table 3.

Because the four outcomes were derived from the same accelerometer recordings and are strongly intercorrelated, the assumption of independence underlying the procedure is only approximately satisfied; the adjusted values should therefore be regarded as a sensitivity analysis rather than as a formal error-rate guarantee.

As in Table 3, the direction indicating a favorable trajectory differs between columns: in the three PA columns a positive coefficient denotes a slower decline in PA, whereas in the SB column a positive coefficient denotes a greater increase in SB.

Abbreviations: BMI, body mass index; mMRC, modified Medical Research Council dyspnea scale; IC, inspiratory capacity; FVC, forced vital capacity; FEV1.0, forced expiratory volume in one second; %pred, % of predicted value; 6MWD, 6-minute walk distance; 6MWT, 6-minute walk test; SpO_2, percutaneous oxygen saturation; FBG, fasting blood glucose; HbA1c, hemoglobin A1c; RBC, red blood cell count; Hb, hemoglobin; BNP, brain natriuretic peptide; Alb, albumin; HADS, hospital anxiety and depression scale; PA, physical activity; METs, metabolic equivalents; SB, sedentary behavior.
